# Computing Health Quality Measures Using Informatics for Integrating Biology and the Bedside

**DOI:** 10.2196/jmir.2493

**Published:** 2013-04-19

**Authors:** Jeffrey G Klann, Shawn N Murphy

**Affiliations:** ^1^Laboratory of Computer ScienceMassachusetts General HospitalBoston, MAUnited States; ^2^Research ComputingPartners Healthcare System, Inc.Boston, MAUnited States; ^3^Harvard Medical SchoolBoston, MAUnited States

**Keywords:** medical informatics, healthcare quality assessment, reimbursement, incentive, systems integration, database management systems

## Abstract

**Background:**

The Health Quality Measures Format (HQMF) is a Health Level 7 (HL7) standard for expressing computable Clinical Quality Measures (CQMs). Creating tools to process HQMF queries in clinical databases will become increasingly important as the United States moves forward with its Health Information Technology Strategic Plan to Stages 2 and 3 of the Meaningful Use incentive program (MU2 and MU3). Informatics for Integrating Biology and the Bedside (i2b2) is one of the analytical databases used as part of the Office of the National Coordinator (ONC)’s Query Health platform to move toward this goal.

**Objective:**

Our goal is to integrate i2b2 with the Query Health HQMF architecture, to prepare for other HQMF use-cases (such as MU2 and MU3), and to articulate the functional overlap between i2b2 and HQMF. Therefore, we analyze the structure of HQMF, and then we apply this understanding to HQMF computation on the i2b2 clinical analytical database platform. Specifically, we develop a translator between two query languages, HQMF and i2b2, so that the i2b2 platform can compute HQMF queries.

**Methods:**

We use the HQMF structure of queries for aggregate reporting, which define clinical data elements and the temporal and logical relationships between them. We use the i2b2 XML format, which allows flexible querying of a complex clinical data repository in an easy-to-understand domain-specific language.

**Results:**

The translator can represent nearly any i2b2-XML query as HQMF and execute in i2b2 nearly any HQMF query expressible in i2b2-XML. This translator is part of the freely available reference implementation of the QueryHealth initiative. We analyze limitations of the conversion and find it covers many, but not all, of the complex temporal and logical operators required by quality measures.

**Conclusions:**

HQMF is an expressive language for defining quality measures, and it will be important to understand and implement for CQM computation, in both meaningful use and population health. However, its current form might allow complexity that is intractable for current database systems (both in terms of implementation and computation). Our translator, which supports the subset of HQMF currently expressible in i2b2-XML, may represent the beginnings of a practical compromise. It is being pilot-tested in two Query Health demonstration projects, and it can be further expanded to balance computational tractability with the advanced features needed by measure developers.

## Introduction

### Background and Significance

In 2010, the US Congress enacted a 10-year, $27 billion dollar incentive program to promote adoption and meaningful usage of electronic health record systems (EHRs) [1]. The government’s vision is that this will lead to a “learning health system” in which health care data can be dynamically aggregated and analyzed for applications such as research, population health measurement, and disease surveillance [2]. The Meaningful Use (MU) program is being rolled out in three stages, each of which expands the definition of what is considered meaningful usage. Stage 1 of Meaningful Use (MU1) focused on capturing structured, coded data, but Stages 2 and 3 (MU2 and MU3) move toward the larger goal of a learning health system, through a focus on population health improvement enabled by Clinical Quality Measures (CQMs)[3].

MU1 involved a small number of computationally simple CQMs [4]. The final rules for MU2 [5,6] require between 9 and 24 CQMs from a menu of 93 [5], many of which are more complex than the MU1 measures, involving test results, deeper computation, and many more elements. The MU3 Request for Comment (RFC) discusses CQMs as the basis of population health management dashboards and introduces the idea that an EHR might need to respond to arbitrary (not pre-defined) CQMs [7]. The goal appears to be dynamic, distributed population health queries [8], enabled by standards and technologies from the Query Health initiative [9-11].

Query Health was convened by the Office of the National Coordinator for Health Information Technology (ONC) in 2011 to develop a standard approach for distributed population health queries. Query Health has defined a standards-based methodology using the Health Level 7 (HL7) Health Quality Measures Format (HQMF) for queries and the Quality Reporting Document Architecture (QRDA) for responses. Additionally, Query Health has developed a reference implementation using their selection of three best-of-breed technological components [9,10].

Query Health has three current pilots, which each use parts of this reference implementation [12]. Two of the pilots are in cooperation with a Department of Health (New York City and Massachusetts) for disease monitoring and surveillance. In the third, the FDA will use Query Health for medication safety surveillance as part of their Mini-Sentinel project. Mini-Sentinel has predominantly used administrative data, and the FDA is evaluating the increased utility of using clinical information.

Two of the pilots use the Informatics for Integrating Biology and the Bedside (i2b2) analytical platform for processing HQMF. i2b2 is a flexible, componentized clinical analytics and data warehousing platform that now enjoys widespread adoption as a research data repository, warehousing clinical data alongside the EHR at over 80 sites nationwide. It is part of an NIH-funded center charged with developing a national computational infrastructure for biomedical computing [13]. i2b2 has underpinned many studies that have discovered new knowledge about disease and its genetic bases (eg, [14]). As part of the Query Health Reference implementation, i2b2 now needs to support composition and consumption of HQMF.

HQMF is becoming the lingua franca for defining population health queries. A draft standard of HQMF was released by HL7 in 2010 [15] and was adopted by the National Quality Forum (NQF) and Query Health. In 2011, the NQF converted 113 of its CQMs to HQMF format (called eMeasures) [16], and now they, with the Centers for Medicare and Medicaid Services (CMS), have published the 93 CQMs used in MU2 as eMeasures [17]. The first HQMF draft standard expired in March 2012, and a second draft standard has been developed jointly by Query Health and HL7 and is in ballot at the time of this writing [18]. This release (HQMFr2) is vastly more computable, more readable, and less bulky. In addition to its usage by Query Health, the NQF is taking steps to re-tool their HQMFr1 eMeasures into HQMFr2. They are balloting an HQMF Implementation Guide based on their Quality Data Model (QDM) [19], which defines a comprehensive set of health care data elements and associated states and attributes (eg, medications can have dose and frequency). This HQMF QDM Implementation Guide will enforce a standard methodology for eMeasure re-tooling. Allscripts is planning an eMeasures interpreter for MU2, well in advance of any federal incentive for handling HQMF [12]. Nonetheless, the MU3 RFC does indicate that mandated electronic processing of HQMF is on the horizon [7].

Throughout the remainder of this manuscript, HQMF will refer to the revised HQMFr2 developed for the ballot now in progress, which is used by the Query Health pilots. Areas under active discussion in the ballot are noted.

### Objective

Our goal is to integrate i2b2 with the Query Health HQMF architecture, to prepare for other HQMF use cases (such as MU2 and MU3), and to articulate the functional overlap between i2b2 and HQMF. Therefore, we analyze the structure of HQMF, and then we apply this understanding to HQMF computation on the i2b2 clinical analytical database platform. Specifically, we develop a translator between two query languages, HQMF and i2b2, so that the i2b2 platform can compute HQMF queries.

## Methods

### Understanding HQMF

HQMF is a language for defining quality measures expressed as logical combinations of clinical variables, intended to return an aggregate count or percentage. For example, HQMF can express, in a computable manner, the following query: “the number of diabetes patients who have had a hemoglobin A1C test greater than 9% in the past year”.

HQMF is derived from the HL7 v3 Reference Implementation Model (RIM), which is also the basis for Clinical Document Architecture (CDA) documents. Therefore CDA bears some resemblance to HQMF. Articulating all of the details of the HQMF format is beyond the scope of this article. Our purpose here is to highlight the structure of HQMF necessary to develop an implementation. For complete documentation, refer to the balloted HQMFr2 [18], the balloted HQMF QDM Implementation Guide [19], and the Query Health HQMF Implementation Guide [20].

We present HQMF through two examples, both based on the NQF0059 measure to detect poorly controlled diabetes [21]. In the first example, we outline the structure of an HQMF query for basic measurement. The second example highlights additional complexities needed to support the more challenging nuances of HQMF. (Note that both examples are illustrative but not complete HQMF; for compact presentation, some XML elements are not included. These include HL7 XML bulk, HQMF headers, and some elements described in the text that would not fit in the figure.)

#### Example 1: Basic HQMF

**Figure 1:**
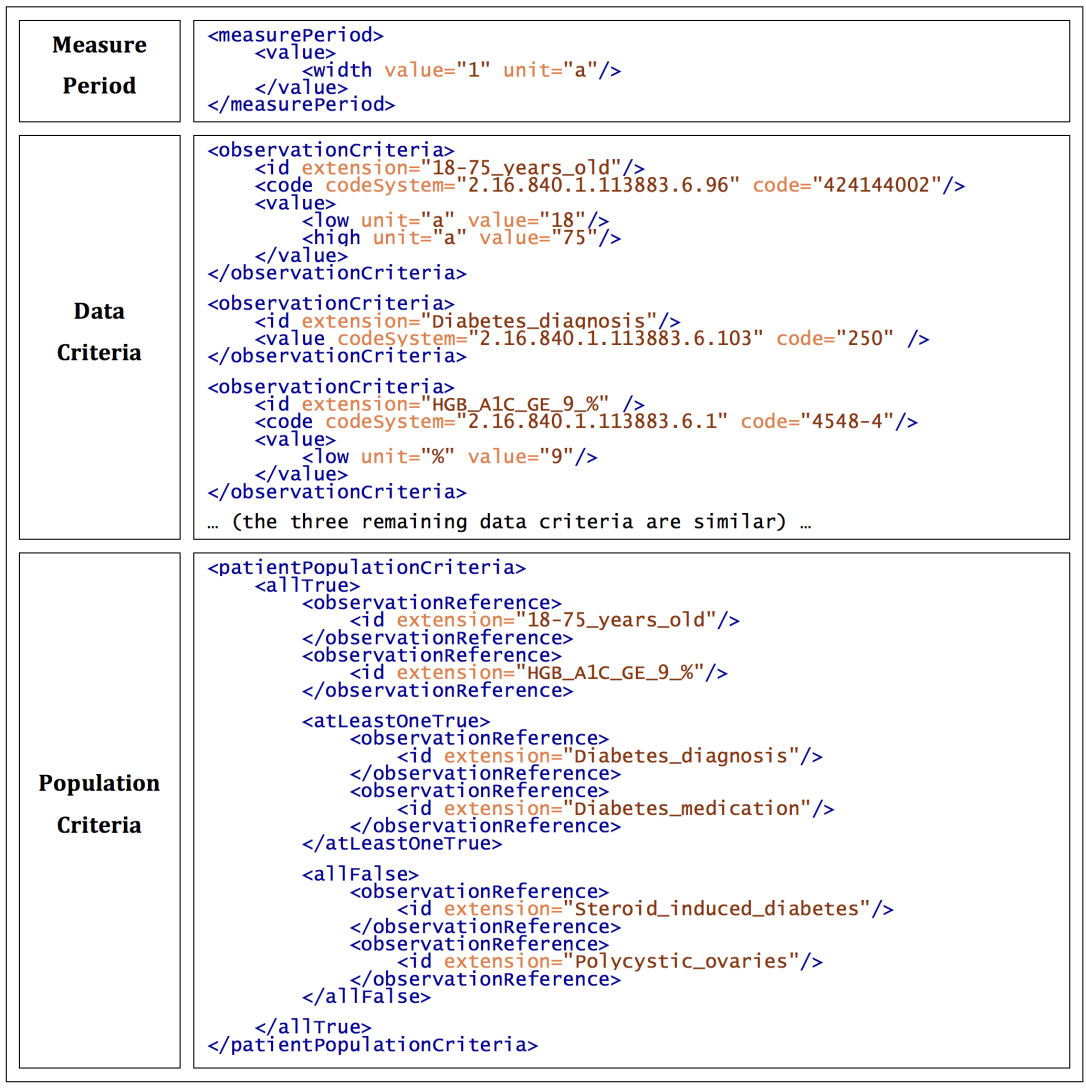
The anatomy of a basic HQMF query (shown here: a portion of a simplified NQF0059 diabetes measure in HQMF).

The anatomy of a basic HQMF query is shown in Figure 1. This simplification of NQF0059 defines the following query: “Find all patients seen in the past year between 18-75 who have, during that year, had either a diabetes diagnosis or have taken a diabetes medication, have had an abnormally high HgbA1c test result, and have not been documented to have polycystic ovaries or steroid induced diabetes.” Although this is a simplification, it is a meaningful quality measure. Figure 1 shows the three basic components of an HQMF document: Measure Period, Data Criteria, and Population Criteria.

##### Measure Period

Measure Period defines the time period the overall query covers, which in this case is 1 year (value *1*, unit *a*). Individual criteria can further restrict the time search.

##### Data Criteria

The Data Criteria section defines the clinical variables in the query. Each variable is a *criteria* entry, the types of which correlate with the *act* classes in CDA. Three data criteria are shown in Figure 1. Here, all criteria are observation criteria, but criteria also exist for encounters, procedures, medications (supplied and administered), and general acts. Each criterion most commonly defines a code, value, and an id. *id* is used to reference the criterion in the population criteria section via the *extension* attribute. *code* defines what is being measured, and *value* defines its result. Note that what is a code and what is a value is not always intuitive. For example, the diagnosis of diabetes is a value (where the optional code refers to the “problem type”, eg, diagnosis, complaint, etc.), but the HgbA1c test is a code and its result is a value.

*Code* can be either a coded value or value set. A coded value is a numeric code and a code system (eg, SNOMED, LOINC, ICD-9) represented by an HL7-registered Object Identifier (OID). A value set is a set of coded values referred to by a single OID. The coded values are OR’d together and are convenient shorthand when defining measures where an observation like “diabetes” could be recorded as multiple specific codes (eg, various ICD-9, ICD-10, or SNOMED codes). At the time of this writing, the National Library of Medicine has begun hosting a small number of value sets at their Value Set Authority Center (VSAC) repository [22,23].

*Value* can be any HL7 data type. When it is not a coded value, it is frequently one of: (1) a numeric value, when a specific value is desired, such as an HgbA1c of exactly 9%; or (2) an interval, seen in the age range and HgbA1c test in Figure 1.

##### Population Criteria

The Population Criteria section defines the calculations on these data elements needed to compute the quality measure. Figure 1 defines one population through a series of nested lists. Each list contains references to data criteria (through the *extension* attribute) as well as other lists. Each list begins with one of six logical “combining operators”, three of which are shown in the figure. The others are *atLeastOneFalse*, *onlyOneTrue*, and *onlyOneFalse*.

#### Example 2: Advanced HQMF

**Figure 2:**
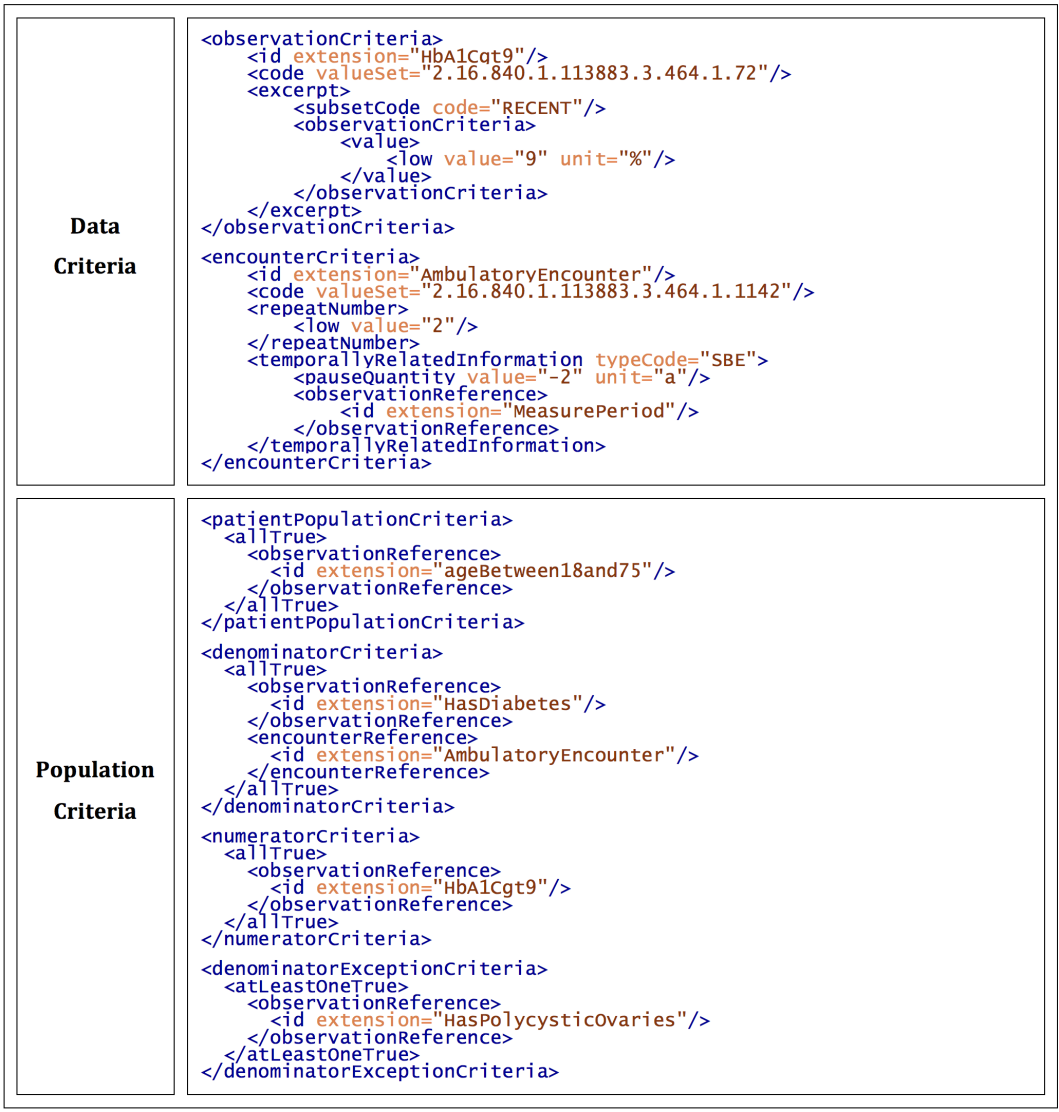
The anatomy of a full-featured HQMF query (shown here: a portion of a simplified NQF0059 diabetes measure in HQMF, highlighting advanced computational features of HQMF).

Example 1 presented a pedagogical simplification of the NQF0059 measure. In this second example, we introduce additional features present in the actual measure. The updated query (with changes italicized) is: “Find all the patients who: *were seen at least twice within the two years before the end of the measure period*; are between 18 and 75; and within the measure period have had either a diabetes diagnosis or have taken a diabetes medication, had an abnormally high value for *their most recent* HgbA1c test result, and are not documented to have polycystic ovaries or steroid induced diabetes.” Note that this still is not quite as complex as the actual measure, but it does use all of the HQMF features present in that measure. HQMF corresponding to the two modified data criteria and the new population criteria section can be seen in Figure 2. This uses the following additional HQMF features.

##### Value Sets

The *code* elements in the data criteria now reference value set OIDs, rather than defining a coded value. These are published by the NQF, and some are available for download from the NLM’s VSAC repository [23].

##### Excerpting

Excerpting applies a filter to a data criterion and reports a summary value of the filtered results (eg, first, last, largest, smallest). This can been seen in the first data criterion in Figure 2. It is reminiscent of the HgbA1c criterion from Figure 1, but it now selects only the most recent HgbA1c test result with a value greater than 9%. This is done by wrapping the *value* element with an *excerpt* element with a subset code of “RECENT”. This means, “find all HgbA1c test results within the measure period, filter out any results not greater than 9%, and report the most recent.”

##### Counting Repetitions

The second data criterion in Figure 2 defines ambulatory encounters using an NQF value set. The *repeatNumber* element specifies that at least two ambulatory encounters must have occurred.

###### Temporal Relationships

Also in the second data criterion in Figure 2, the *temporallyRelatedInformation* element specifies that these encounters must have occurred within 2 years (*pauseQuantity*) of the end (*typecode=SBE—*“starts before end”) of the measure period (*observationReference*). Multiple temporal relationships can occur within a single criterion, and 17 types of relationships are defined. In addition to the measure period, temporal relationships can reference other data criteria.

###### Multiple Population Criteria

Figure 2 has four population criteria: an initial patient population (all people between 18 and 75), a denominator (those with diabetes), a numerator (those with abnormal HgbA1c values), and exceptions (those with polycystic ovaries). This multipopulation approach has two purposes. One, it allows measurement results to be reported as a percentage: numerator divided by denominator. Two, smaller population components are more modular. This has an organizational advantage for measure developers, but it also could speed computation. If multiple queries with the same denominator and exceptions are run with different numerators, the denominator can be computed just once.

#### Completing an HQMF Implementation

These two examples cover all the major features in NQF0059. However, to support very complex measures, HQMF implementations should also include the following features.

##### Stratifiers

Normally, results are reported as one aggregate number, but stratifiers allow reporting to be broken down by other criteria (eg, zipcode or gender).

##### Denominator Exclusions

An additional population group, this describes criteria for patients who should be excluded from the denominator only if they do not meet numerator criteria (for example, the measure could be modified to not penalize practices for uncontrolled diabetics who are high risk, eg, who have been to the emergency room at least five times in the past year).

##### Calculations on Continuous Variables

This might include, for example, the average emergency department wait time.

##### Modifiers

Data criteria can have other modifiers beyond those discussed here. Some of these are simply data elements, eg, the clinician interpretation code for a vital sign goes in an *interpretationCode* element. Others, such as details of medication route, admitting physician, or problem status require more complex XML structure using RIM data elements. Implementation Guides (IGs) will define how such elements should be expressed. The only available resources at the time of this writing are the Query Health IG draft and standards developer Keith Boone’s blog [20,24]. (The QDM IG leaves criteria-level templates to a future release.) Some modifier structure can be inferred from CDA IGs, though portability across implementations cannot be guaranteed until a complete set of templates in an HQMF IG are available.

##### Missing Information

Values can explicitly be “no information”, allowing special behavior when information is missing.

##### Specific Occurrences

Under active discussion is a canonical representation for multiple observations that must be temporally related to a specific occurrence of another observation (eg, multiple observations referring to a single encounter).

### Understanding i2b2

i2b2 consists of a flexible relational data model and a somewhat more restricted set of Web services.

The data model stores observations as “facts” associated with a date, patient identifier, and encounter identifier. Each fact has a key that follows a hierarchical structure (eg, “Diagnosis\Diabetes Mellitus\Diabetes With Ketoacidosis”) and a basecode that defines the source code and coding system (eg, “ICD9:250.6”). Optional modifier facts can supplement each primary fact (eg, “admit diagnosis”). Facts can have a value, stored in numeric or text formats. The set of possible facts (the ontology) are user-defined, though several standard ontologies exist.

The web services define an easy-to-understand query language expressed in an XML format. Queries are built in a web-based query tool and executed as highly optimized SQL statements by a “data repository” web service. A query consists of items dragged into a set of panels. The items in each panel are logically OR’d together, and all panels are logically AND’d. The NOT operation can optionally be applied to a panel. Each item can also have date constraints, though the only other temporal constraint currently supported is “all items in panel must be in the same encounter.” Results can be stratified on any observation (eg, age, race, gender), and queries can be combined through query-within-a-query (eg, a previous query definition can be dragged into a panel), which allows deep nesting and more complex combinations of elements. Furthermore, ontology items can have arbitrary SQL statements embedded, for added flexibility.

This query XML is logically equivalent to a subset of HQMF, and it is therefore possible to translate between these two formats, provided that queries are restricted to this subset.

## Results

We first illustrate our translation method between i2b2-XML and HQMF by describing the conversion of Example 1 to an i2b2 query. Next, we analyze the HQMF subset supported by i2b2-XML, and we describe a web service we have implemented for i2b2 to both generate and consume HQMF conforming to this subset.

### Example 1 As i2b2

**Figure 3:**
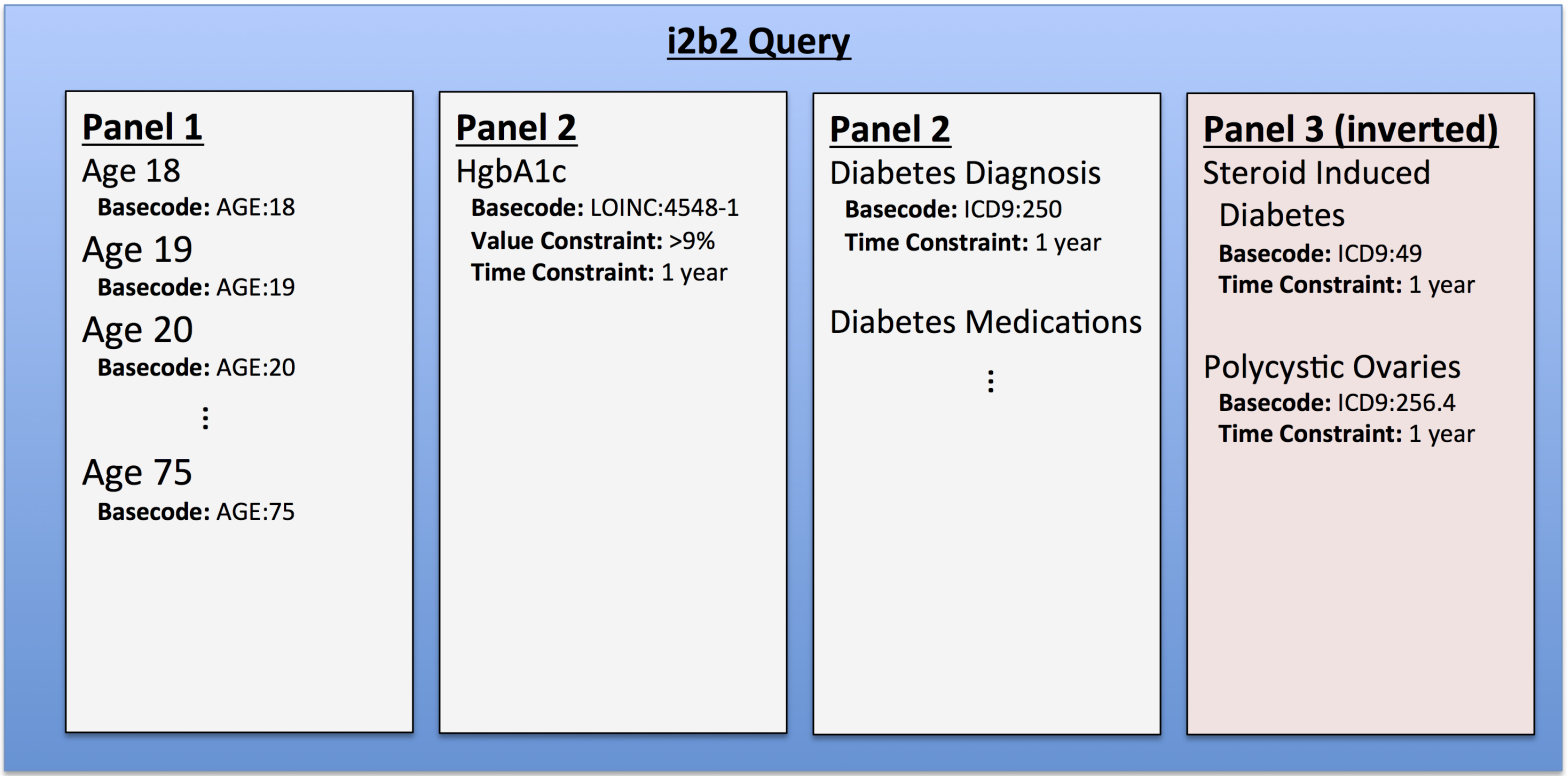
The HQMF from Figure 1 converted to i2b2 query panels.

Figure 3 shows an i2b2 query version of Figure 1, displayed as i2b2 panels. The conversion of each element from HQMF to i2b2-XML is as follows.

#### Data Criteria Conversion

First, each data criterion is converted to an i2b2 basecode. For age ranges (panel 1), age is expanded to the OR of every age in the range using the AGE basecode. (There is no standard terminology to encode age ranges; this is the only case of specialized logic for a particular data element.) For all other values, an OID-to-i2b2 lookup table determines the basecode, which is implementation-configurable.

Second, i2b2 basecodes are sent to the i2b2 ontology cell for conversion to associated i2b2 keys. An i2b2 key represents a position in the ontology tree, so the ICD diagnosis codes in panels 2 and 3 include not only the listed basecode (eg, ICD9:250) but also all subcodes (eg, ICD9:250.xx). This nonstandard HQMF interpretation allowed us to specify an implicit diabetes value set with single coded value, prior to standardization of NQF value sets. Therefore, we feel this behavior (which will normally affect only ICD codes) is useful at the present time, and we will revisit it once the NLM VSAC is more complete.

Third, value constraints are added, such as the numeric interval “>9%” for HgbA1c.

Fourth, time constraints are added. When no specific time constraint is specified, a time constraint is added to match the measurement period.

#### Population Criteria Conversion

For *allTrue,* every item or list following is placed in a separate panel. For *atLeastOneTrue*, every item following is placed in the same panel. For *allFalse,* every item following is placed in the same panel, which is set to the *exclude* type (ie, the NOT operation is performed on each item in the panel).

### HQMF i2b2 Translator

HQMF features currently supported by i2b2-XML are shown in Table 1. For the first version of the translator, we targeted a subset of these features that cover the functionality of the Shared Health Research Information Network (SHRINE), a distributed version of i2b2 in use around the country for performing distributed population queries [25].

**Figure 4:**
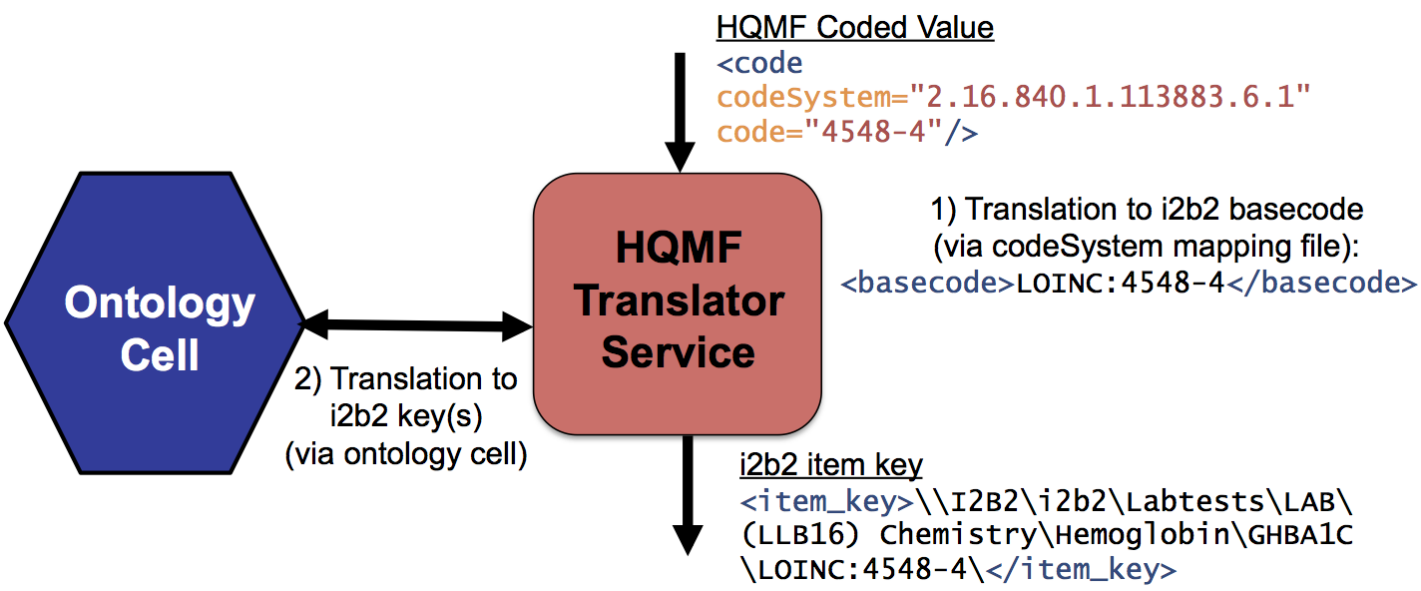
Translation from an HQMF coded value to i2b2 item key(s).

We have implemented a bidirectional translator that can convert between HQMF-coded values and i2b2 keys (see Figure 4). For translation from HQMF, the basecode lookup method in the previous example is used. For translation to HQMF, a reverse lookup retrieves the basecode from the key. For consistency, all child nodes of the key are by default included (eg, \\I2B2\Demographics\Zipcodes\Alabama is augmented by an item for every zipcode in Alabama). The translator further supports result values, date constraints, repetition counting (at the panel level), and a single population with multiple *atLeastOneTrue* and *allFalse* groups inside an *allTrue* group.

Stratifiers, excerpts, and features requiring query-within-a-query are not currently supported. Stratifiers were a low priority for the Query Health pilots, so they have been tabled. Query-within-a-query features are waiting on the more portable implementation in i2b2-XML planned for i2b2’s next release. Excerpts will be supported in the future through ontology definitions containing custom SQL code.

Modifiers and value sets are partially supported, but they also require an appropriate HQMF i2b2 ontology (containing approved value sets and supported modifiers). Dr. Michael Buck in the New York Department of Health is developing this ontology, which is an expression of CDA and QDM data elements in i2b2. We have tested his ontology’s ability to support HQMF modifiers and value sets by successfully implementing support for health care providers and clinician interpretation codes and by testing conversion of his NQF0059-compatible diabetes value set. When completed, this ontology will significantly aid i2b2 in consuming complex CQMs.

To validate the translator’s accuracy, we developed four query libraries in i2b2-XML, one for each supported ontology configuration of the translator (SHRINE, i2b2 default, Dr. Buck’s terminology, and Beth Israel Deaconess Medical Center). Each library utilizes all features of the translator (see Table 1 and the discussion above). As we developed the translator, we (the authors or a member of the Query Health Reference Implementation team) periodically translated each library to HQMF and back to i2b2-XML. We visually inspected the HQMF and i2b2-XML to verify they were semantically equivalent to the original (because the translator inserts basecodes for child nodes, syntactic equivalence might not occur). We also had access to databases of (fake) test patients for all but the Beth Israel configuration, so we also ran both the original and post-translation i2b2-XML against that and verified the result was the same. Finally, we periodically translated and validated an HQMFr2 version of NQF0059 available in Query Health’s repository. No other publicly available source of HQMFr2 presently exists (though this will rapidly change once HQMFr2 is through ballot).

**Figure 5:**
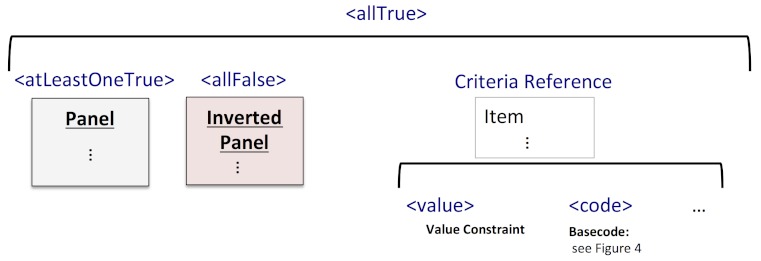
The transformation process from HQMF to i2b2-XML.

Our translator supports a superset of SHRINE’s features, and it is able to generate and process HQMF corresponding to the i2b2-XML feature set in Table 1 except as noted above. It can consume HQMF from other sources, as long as it is restricted to this feature set. The transformation process is summarized visually in Figure 5, with an illustration of i2b2 panels being built from the population criteria. When a criteria reference is encountered, an i2b2 item is inserted using information from the data criteria element. Supported HQMF elements include *value*, *code*, temporal constraints on the measure period, and some modifiers and value sets. The reverse translation follows the diagram in reverse (data criteria are generated from i2b2 items, and population criteria are built from the panel layout).

The translator is by default configured for diagnoses, labs, procedures, medication administration events, and the demographics in SHRINE (Age, Gender, Language, Marital Status, and Race and Ethnicity). Non-SHRINE ontologies are supported through a configuration file. The translator is open source and presently available from the Query Health repository [26]. It will likely be included in a future version of i2b2. The translator is implemented in Java and XSL as a Jersey servlet that runs within the i2b2 JBoss stack. A service to connect i2b2 to a Query Health-conformant, HQMF-based distributed query engine is being completed at the time of this writing and will also be available as an open-source project.

**Table 1:** HQMF features and their equivalence in the i2b2 XML query language (features not fully equivalent are italicized; note that some supported features are ontology-dependent).

	HQMF	i2b2-XML
**Data Criteria**		
	Coded values, with optional result value	Facts (looked up by basecode)
	Value sets, with optional result value	Facts (requires an ontology with value set OIDs as basecodes)
	Modifiers	Facts (requires an appropriate ontology)
	Excerpts	Facts (requires ontology definitions)
	Repetition counting	*Supported at the panel level.*
	Temporal relationships	*Date constraints (such as the measure period) only; others to be supported in summer 2013.*
**Population Criteria**		
	Multiple populations	*Populations are constructed separately and can be combined with query-within-query.*
	Calculations on continuous variables	*Supported conceptually through patient-data objects and client-side analysis, but a plugin would be needed to perform HQMF server-side calculations.*
	allTrue	The ANDing of all panels.
	atLeastOneTrue	The ORing of elements in a panel.
	allFalse	An inverted panel
	atLeastOneFalse	A set of inverted panels, each consisting of a previous query.
	onlyOneTrue	Not supported
	onlyOneFalse	Not supported
	Nested “combining operators”	atLeastOneTrue and allFalse groups inside an allTrue
	Stratifiers	Supported (but requires server configuration).

## Discussion

We have achieved significant interoperability between HQMF and i2b2 using only transformations of the two XML languages. Our web service successfully transforms queries bidirectionally between i2b2-XML and HQMF, supporting a superset of all queries possible in SHRINE. This HQMF translator has been integrated into the reference implementation of Query Health and is currently being used at two Query Health pilot sites. The first, through the New York State Department of Health, utilizes the bidirectional translator with Dr. Buck’s ontology, allowing researchers to create custom HQMF queries and execute them at i2b2 sites throughout New York. The second pilot, at the Beth Israel Deaconess Medical Center, connects their i2b2 instance (using the SHRINE ontology) to the FDA Mini-Sentinel network and demonstrates the FDA’s ability to send custom HQMF queries to i2b2 instances. i2b2 1.7, to be released in summer 2013, will extend the i2b2-XML language to support a larger subset of HQMF, including improved query-within-query and new temporal constraints. We hope this, along with Dr. Buck’s ontology, will give us the tools needed to compute CQMs for MU2 and MU3 using the XML translation approach.

HQMF is an elegant but complex and challenging query language. It separates the definition of data elements from the logical operations used to combine them, and the format for clinical variables is similar to CDA, making basic understanding less difficult for HL7 developers. However, even the partial support we have achieved has been a massive effort. The work involved was more than 6 months of FTE time by an experienced software architect, and we had a preexisting sample transform from standards developer Keith Boone, support from Query Health team members, and the i2b2 analytical database system with a schema-defined XML language. Even with i2b2 1.7 and Dr. Buck’s ontology, full translator support will involve much additional effort. Although future implementers will have both the advantage of our experiences and an open-source, HQMF-enabled version of i2b2, software development groups should carefully consider the required effort. Furthermore, the current HQMF specification allows some query constructions that are computationally challenging. Multiple time relationships on a single criterion, the possibility of nesting time relationships and excerpts, arbitrarily deep nesting of population criteria groups, and the fact that not all population criteria operators are equivalent to logical operators suggests to us that running very complex queries could tax even powerful servers. Also, HQMF allows unrealistic query constructions. In particular, supporting 16 temporal relationships between criteria does not reflect the limited temporal granularity we have seen in existing clinical data warehouses. Finally, HQMF has limited ability to specify behavior when data are missing or noisy. Revisions to implementation guides and the HQMF standard will be needed to address these issues. Some of these difficulties (eg, reducing complexity by limiting nesting in data criteria) are being discussed in the current ballot reconciliations.

### Conclusion

HQMF is a powerful language for developing and computing measures of population health. Despite complexity concerns, we believe this format can be supported, which will be important in stage two of meaningful use and possibly required in stage three. HQMF is also a key component in Query Health and will likely play important roles in other health informatics initiatives. We have created a fully bidirectional HQMF translator, which converts between HQMF and i2b2-XML formats, supporting a superset of SHRINE’s features. This translator is freely available and has been integrated into the reference implementation of Query Health. Areas that we have been unable to implement will either be addressed in the next release of i2b2 or will be brought before appropriate decision-makers and standards developers. Expanding i2b2’s HQMF support through this XML translation approach will allow i2b2 to fully support the requirements of CQM processing in MU. The work lays a foundation for dynamic, distributed queries across diverse clinical systems with disparate data models.

## Abbreviations

CDA: HL7’s Clinical Document Architecture
CMS: Centers for Medicare and Medicaid Services
CQM: clinical quality measure
EHR: electronic health record
FTE: full-time employee
HgbA1c: Hemoglobin A1c, a blood sugar test
HL7: Health Level Seven, a health care standards organization
HQMF(r2): Health Quality Measures Format (revision 2)
i2b2: Informatics for Integrating Biology and the Bedside
ICD9: International Classification of Diseases, 9th Edition
IG: implementation guide
LOINC: Logical Observation Identifiers Names and Codes
MU: Meaningful Use Incentive Program, where MU1, MU2, and MU3 refer to the specific stages of the program.
NLM: National Library of Medicine
NQF: National Quality Forum
NQF0059: NQF CQM 0059, defining poorly controlled diabetes
ONC: Office of the National Coordinator for Health Information Technology
QDM: NQF’s Quality Data Model
RFC: request for comment
RIM: HL7’s reference implementation model
SHRINE: Shared Health Research Information Network
SNOMED: Systematized Nomenclature of Medicine
SQL: Structured Query Language
VSAC: NLM’s Value Set Authority Center
XML: extensible markup language
